# Supplementary figures and images for: Information and decision-making needs of psychiatric patients: the perspective of relatives
Source: PeerJ. 2017 Jul 6;5:e3378. doi: 10.7717/peerj.3378 (PMC5501965; doi:10.7717/peerj.3378)

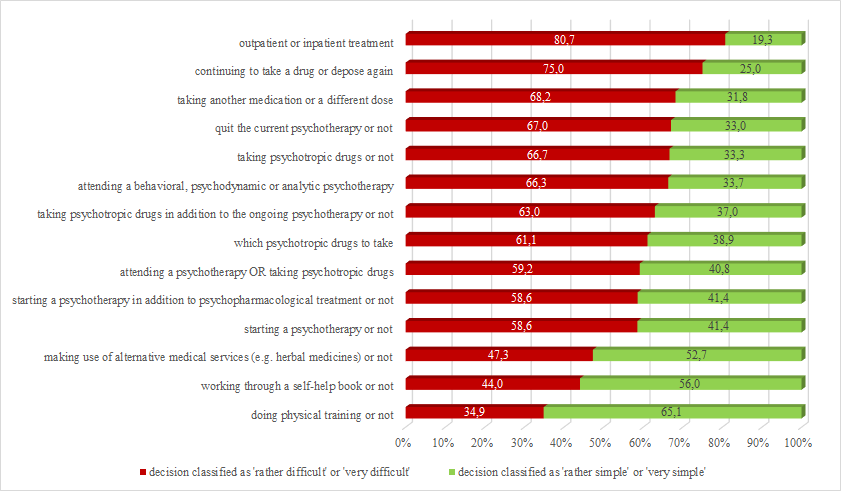

Supplement: Figure S1 — This figure displays difficulty of making a decision as a percentage of those who had actually made the decision ( N = 74–162). [file peerj-05-3378-s001.png]
