# Supplementary material for: Information and decision-making needs of psychiatric patients: the perspective of relatives
Source: PeerJ. 2017 Jul 6;5:e3378. doi: 10.7717/peerj.3378 (PMC5501965; doi:10.7717/peerj.3378)
Supplement: Table S1 — This table displays the relation between preferred role and decisional conflict for those who had actually made the decision (N = 74–162). [file peerj-05-3378-s002.docx]

|  |  | **Preferred role in decsion-making** | | |  |  |  |  |  |
| --- | --- | --- | --- | --- | --- | --- | --- | --- | --- |
|  | **decision classified as…** | **active (affected person alone)** | **shared (affected person & health practinioner)** | **passive (health practitioner alone)** | **N** | **Chi²** | **df** | **exact significance (two-tailed)** | **Phi** |
| outpatient or inpatient treatment | difficult | 22 | 88 | 20 | 130 | 2.55 | 2 | .272 | .126 |
|  | simple | 9 | 17 | 5 | 31 |  |  |  |  |
| taking psychotropic drugs or not | difficult | 21 | 72 | 15 | 108 | 1.82 | 2 | .437 | .106 |
|  | simple | 9 | 33 | 12 | 54 |  |  |  |  |
| which psychotropic drugs to take | difficult | 14 | 59 | 15 | 88 | 0.20 | 2 | .966 | .037 |
|  | simple | 9 | 39 | 8 | 56 |  |  |  |  |
| taking another medication or a different dose | difficult | 14 | 57 | 19 | 90 | 4.20 | 2 | .125 | .178 |
|  | simple | 9 | 30 | 3 | 42 |  |  |  |  |
| continuing to take a drug or depose again | difficult | 16 | 68 | 21 | 105 | 2.71 | 2 | .285 | .139 |
|  | simple | 9 | 22 | 4 | 35 |  |  |  |  |
| starting a psychotherapy or not | difficult | 19 | 57 | 13 | 89 | 0.29 | 2 | .864 | .044 |
|  | simple | 12 | 40 | 11 | 63 |  |  |  |  |
| quit the current psychotherapy or not | difficult | 12 | 36 | 11 | 59 | 0.40 | 2 | .860 | .067 |
|  | simple | 6 | 16 | 7 | 29 |  |  |  |  |
| attending a behavioral, psychodynamic or analytic psychotherapy | difficult | 17 | 42 | 10 | 69 | 4.81 | 2 | .096 | .215 |
|  | simple | 3 | 23 | 9 | 35 |  |  |  |  |
| taking psychotropic drugs in addition to the ongoing psychotherapy or not | difficult | 10 | 50 | 8 | 68 | 2.64 | 2 | .261 | .156 |
|  | simple | 7 | 24 | 9 | 40 |  |  |  |  |
| starting a psychotherapy in addition to psychopharmacological treatment or not | difficult | 14 | 42 | 12 | 68 | 0.35 | 2 | .859 | .055 |
|  | simple | 8 | 32 | 8 | 48 |  |  |  |  |
| attending a psychotherapy OR taking psychotropic drugs | difficult | 5 | 29 | 8 | 42 | 4.31 | 2 | .123 | .246 |
|  | simple | 9 | 17 | 3 | 29 |  |  |  |  |
| working through a self-help book or not | difficult | 4 | 27 | 6 | 37 | 3.64 | 2 | .181 | .208 |
|  | simple | 13 | 28 | 6 | 47 |  |  |  |  |
| making use of alternative medical services (e.g. herbal medicines) or not | difficult | 4 | 26 | 5 | 35 | 3.25 | 2 | .224 | .210 |
|  | simple | 11 | 24 | 4 | 39 |  |  |  |  |
| doing physical training or not | difficult | 9 | 33 | 3 | 45 | 1.26 | 2 | .568 | .099 |
|  | simple | 16 | 57 | 11 | 84 |  |  |  |  |
