# Supplementary material for: Information and decision-making needs of psychiatric patients: the perspective of relatives
Source: PeerJ. 2017 Jul 6;5:e3378. doi: 10.7717/peerj.3378 (PMC5501965; doi:10.7717/peerj.3378)
Supplement: Table S2 — This table displays the relation between accordance (between actual and preferred role) and decisional conflict for those who had actually made the decision (N = 74–162). [file peerj-05-3378-s003.docx]

|  | **decision classified as…** | **accordance between preferred and actual role** | **discrepancy between preferred and actual role** | **N** | **chi²** | **df** | **exact significance (two-tailed)** | **phi** |
| --- | --- | --- | --- | --- | --- | --- | --- | --- |
| outpatient or inpatient treatment | difficult | 64 | 66 | 130 | 1.11 | 1 | .322 | .083 |
|  | simple | 12 | 19 | 31 |  |  |  |  |
| taking psychotropic drugs or not | difficult | 53 | 55 | 108 | 0.01 | 1 | 1.000 | -.009 |
|  | simple | 27 | 27 | 54 |  |  |  |  |
| which psychotropic drugs to take | difficult | 44 | 44 | 88 | 0.18 | 1 | .734 | .035 |
|  | simple | 26 | 30 | 56 |  |  |  |  |
| taking another medication or a different dose | difficult | 43 | 47 | 90 | 0.00 | 1 | 1.000 | .001 |
|  | simple | 20 | 22 | 42 |  |  |  |  |
| continuing to take a drug or depose again | difficult | 52 | 53 | 105 | 0.01 | 1 | 1.000 | .008 |
|  | simple | 17 | 18 | 35 |  |  |  |  |
| starting a psychotherapy or not | difficult | 49 | 40 | 89 | 2.20 | 1 | .188 | .120 |
|  | simple | 27 | 36 | 63 |  |  |  |  |
| quit the current psychotherapy or not | difficult | 32 | 27 | 59 | 0.69 | 1 | .498 | .088 |
|  | simple | 13 | 16 | 29 |  |  |  |  |
| attending a behavioral, psychodynamic or analytic psychotherapy | difficult | 31 | 38 | 69 | 1.39 | 1 | .300 | -.115 |
|  | simple | 20 | 15 | 35 |  |  |  |  |
| taking psychotropic drugs in addition to the ongoing psychotherapy or not | difficult | 36 | 32 | 68 | 1.10 | 1 | .324 | .101 |
|  | simple | 17 | 23 | 40 |  |  |  |  |
| starting a psychotherapy in addition to psychopharmacological treatment or not | difficult | 42 | 26 | 68 | 4.57 | 1 | .039 | .198 |
|  | simple | 20 | 28 | 48 |  |  |  |  |
| attending a psychotherapy OR taking psychotropic drugs | difficult | 21 | 21 | 42 | 1.01 | 1 | .343 | .119 |
|  | simple | 11 | 18 | 29 |  |  |  |  |
| working through a self-help book or not | difficult | 20 | 17 | 37 | 0.01 | 1 | 1.000 | -.013 |
|  | simple | 26 | 21 | 47 |  |  |  |  |
| making use of alternative medical services (e.g. herbal medicines) or not | difficult | 15 | 20 | 35 | 0.53 | 1 | .494 | -.084 |
|  | simple | 20 | 19 | 39 |  |  |  |  |
| doing physical training or not | difficult | 28 | 17 | 45 | 2.12 | 1 | .195 | .128 |
|  | simple | 41 | 43 | 84 |  |  |  |  |
