# Supplementary material for: Information and decision-making needs of psychiatric patients: the perspective of relatives
Source: PeerJ. 2017 Jul 6;5:e3378. doi: 10.7717/peerj.3378 (PMC5501965; doi:10.7717/peerj.3378)
Supplement: Supplemental Information 1 [file peerj-05-3378-s005.pdf]

**Liebe Teilnehmerin, lieber Teilnehmer!**
Wir möchten unser Internetangebot zum Thema „Psychische Erkrankungen“ für Betroffene sowie ihre Angehörigen noch stärker an den Bedürfnissen seiner Nutzerinnen und Nutzer orientieren.
Deshalb führen wir diese Befragung durch.

Die Erhebung der Daten erfolgt anonym. Rückschlüsse auf einzelne Befragungsteilnehmer sind nicht möglich (siehe Datenschutzinformation).
Ihre Teilnahme an der Befragung ist freiwillig. Ihnen entstehen keinerlei Nachteile, wenn Sie nicht teilnehmen möchten oder die Befragung abbrechen. Die Angebote auf www.psychenet.de können Sie jederzeit nutzen.

**Die Befragung dauert ca. 10 Minuten.**

Haben Sie noch weitere Fragen zu dieser Befragung?
Nähere Informationen erhalten Sie unter Information oder durch unsere Ansprechpartnerin.

Vielen Dank, dass Sie an der Befragung teilnehmen. Sie tragen dazu bei, unsere Informationen zu verbessern!

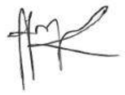

Prof. Dr. Dr. Martin Härter
(Projektleiter)
Aufgrund der besseren Lesbarkeit haben wir uns im Fragebogen nur für die Nennung der männlichen Form (Patient, Arzt, Therapeut etc.) entschieden, selbstverständlich sind hier beide Geschlechter gemeint.

## 2 Einverständniserklärung

Bevor Sie mit der Befragung beginnen, brauchen wir noch Ihr Einverständnis.
Genauere Informationen finden Sie hier.
Hiermit willige ich ein, an der Studie teilzunehmen. Ich habe das Recht, die Befragung jederzeit abzubrechen. Bei einem Abbruch werden alle bereits erhobenen Daten gelöscht.

○ Ich akzeptiere
○ Ich lehne ab

## 3 Angaben zur Person 1

Dieser Fragebogen richtet sich an Angehörige oder Freunde/Bekannte von Menschen mit einer psychischen Erkrankung.
Wenn Sie den Fragebogen als selbst von einer psychischen Erkrankung Betroffener beantworten möchten, klicken Sie auf Fragebogen Betroffene.

**Zunächst möchten wir Sie um ein paar Angaben zu Ihrer Person und Ihren Erfahrungen mit psychischen Erkrankungen bitten:**
**Geschlecht**
○ weiblich
○ männlich
○ anderes

## 4 Angaben zur Person 2

**Welchen höchsten Schulabschluss haben Sie?**
○ noch in der Schule
○ ohne Schulabschluss abgegangen

- Sonderschule/Förderschule
- Haupt-/Volksschulabschluss
- Realschule/Mittlere Reife
- Abschluss der Polytechnischen Oberschule 10. Klasse (vor 1965: 8. Klasse)
- Fachhochschulreife
- erweiterte Oberschule/allgemeine/fachgebundene Hochschulreife/Abitur
- einen anderen Schulabschluss und zwar: (bitte angeben)

**In welcher Partnerschaftsituation leben Sie?**

- Ich habe keine(n) Partner(in)
- Ich habe eine(n) feste(n) Partner(in)
- Ich habe wechselnde Partner(innen)

**In welchem Land sind Sie geboren?**

- in Deutschland
- in einem anderen Land und zwar: (bitte angeben)

## 5 Psychische Erkrankungen

**Mit welcher psychischen Erkrankung haben Sie als Angehöriger oder Freund/Bekannter am meisten Erfahrung?**

Da dieser Fragebogen auf spezielle psychische Erkrankungen abgestimmt ist, ist nur eine Antwort möglich.

- Depression
- Bipolare (manisch-depressive) Störung, Manie
- Somatoforme Störung (Krankheitsangst oder körperliche Beschwerden ohne medizinischen Befund wie z.B. Somatisierungsstörung oder Schmerzstörung)
- Schizophrenie/Psychose
- Essstörung (z.B. Magersucht oder Ess-Brech-Sucht)
- Angststörung
- Schädlicher Alkoholgebrauch oder -abhängigkeit
- andere und zwar: (bitte angeben)
- weiß nicht

**6 Wer im Umfeld**

**Wer in Ihrem Umfeld ist von psychischen Beschwerden betroffen?**
Mehrfachantwort möglich

- ☐ mein(e) Partner(in)
- ☐ meine Mutter/mein Vater
- ☐ meine Tochter/mein Sohn
- ☐ andere Verwandte
- ☐ ein(e) Freund(in)/Bekannte(r)
- ☐ andere und zwar: (bitte angeben) ____

**7 Erfahrungen mit dem Internet**

**Als nächstes geht es um Ihre Erfahrungen mit dem Internet.**
**Wie häufig nutzen Sie das Internet?**

| (fast) täglich | mindestens einmal pro Woche | mindestens einmal im Monat | seltener als einmal im Monat | nie |
|---|---|---|---|---|
| ○ | ○ | ○ | ○ | ○ |

**Wie häufig nutzen Sie das Internet zur allgemeinen Suche nach Informationen über Gesundheitsthemen oder Erkrankungen?**

| (fast) täglich | mindestens einmal pro Woche | mindestens einmal im Monat | seltener als einmal im Monat | nie |
|---|---|---|---|---|
| ○ | ○ | ○ | ○ | ○ |

**Wie häufig nutzen Sie das Internet zur Suche nach Informationen über Depressionen?**

| (fast) täglich | mindestens einmal pro Woche | mindestens einmal im Monat | seltener als einmal im Monat | nie |
|---|---|---|---|---|
| ○ | ○ | ○ | ○ | ○ |

**Wie häufig nutzen Sie das Internet zur Suche nach Informationen über bipolare (manisch-depressive) Störungen bzw. Manien?**

| (fast) täglich | mindestens einmal pro Woche | mindestens einmal im Monat | seltener als einmal im Monat | nie |
|---|---|---|---|---|
| ○ | ○ | ○ | ○ | ○ |

**Wie häufig nutzen Sie das Internet zur Suche nach Informationen über somatoforme Störungen (Krankheitsangst oder körperliche Beschwerden ohne medizinischen Befund wie z.B. Somatisierungsstörung oder Schmerzstörung)?**

| (fast) täglich | mindestens einmal pro Woche | mindestens einmal im Monat | seltener als einmal im Monat | nie |
|---|---|---|---|---|
| ○ | ○ | ○ | ○ | ○ |

**Wie häufig nutzen Sie das Internet zur Suche nach Informationen über Schizophrenie/Psychose?**

| (fast) täglich | mindestens einmal pro Woche | mindestens einmal im Monat | seltener als einmal im Monat | nie |
|---|---|---|---|---|
| ○ | ○ | ○ | ○ | ○ |

**Wie häufig nutzen Sie das Internet zur Suche nach Informationen über Essstörungen (z.B. Magersucht oder Ess-Brech-Sucht)?**

| (fast) täglich | mindestens einmal pro Woche | mindestens einmal im Monat | seltener als einmal im Monat | nie |
|---|---|---|---|---|
| ○ | ○ | ○ | ○ | ○ |

**Wie häufig nutzen Sie das Internet zur Suche nach Informationen über Angststörungen?**

| (fast) täglich | mindestens einmal pro Woche | mindestens einmal im Monat | seltener als einmal im Monat | nie |
|---|---|---|---|---|
| ◯ | ◯ | ◯ | ◯ | ◯ |

**Wie häufig nutzen Sie das Internet zur Suche nach Informationen über schädlichen Alkoholgebrauch oder Alkoholabhängigkeit?**

| (fast) täglich | mindestens einmal pro Woche | mindestens einmal im Monat | seltener als einmal im Monat | nie |
|---|---|---|---|---|
| ◯ | ◯ | ◯ | ◯ | ◯ |

**Wie häufig nutzen Sie das Internet zur Suche nach Informationen über psychische Erkrankungen?**

| (fast) täglich | mindestens einmal pro Woche | mindestens einmal im Monat | seltener als einmal im Monat | nie |
|---|---|---|---|---|
| ◯ | ◯ | ◯ | ◯ | ◯ |

## 8 Filter: Informationssuche

| |
|---|
| Wie häufig nutzen Sie das Internet zur Suche nach Informationen über Depressionen? |
| Wie häufig nutzen Sie das Internet zur Suche nach Informationen über bipolare (manisch-depressive) Störungen bzw. Manien? |
| Wie häufig nutzen Sie das Internet zur Suche nach Informationen über somatoforme Störungen (Krankheitsangst oder körperliche Beschwerden ohne medizinischen Befund wie z.B. Somatisierungsstörung oder Schmerzstörung)? |
| Wie häufig nutzen Sie das Internet zur Suche nach Informationen über Schizophrenie/Psychose? |
| Wie häufig nutzen Sie das Internet zur Suche nach Informationen über Essstörungen (z.B. Magersucht oder Ess-Brech-Sucht)? |
| Wie häufig nutzen Sie das Internet zur Suche nach Informationen über Angststörungen? |
| Wie häufig nutzen Sie das Internet zur Suche nach Informationen über schädlichen Alkoholgebrauch oder Alkoholabhängigkeit? |
| Wie häufig nutzen Sie das Internet zur Suche nach Informationen über psychische Erkrankungen? |

### 8.1 Gründe und Art der Informationen

**Aus welchen Gründen haben Sie im Internet nach Informationen über Depressionen gesucht?**

Mehrfachangaben möglich

- ☐ Die Informationen der behandelnden Ärzte/Psychotherapeuten waren nicht ausreichend.
- ☐ Die Informationen der behandelnden Ärzte/Psychotherapeuten waren schwer zu verstehen.
- ☐ Ich war mit den Informationen der behandelnden Ärzte/Psychotherapeuten nicht einverstanden.
- ☐ Mir wurde vom Arzt/Psychotherapeuten empfohlen, mich über Depressionen zu informieren.
- ☐ Ich bin auf der Suche nach einem Arzt/Psychotherapeuten.
- ☐ Ich wollte mich ganz allgemein über Depressionen informieren.
- ☐ Sonstiges und zwar: (bitte angeben) ______

**Aus welchen Gründen haben Sie im Internet nach Informationen über bipolare Störungen/Manien gesucht?**

Mehrfachangaben möglich

- ☐ Die Informationen der behandelnden Ärzte/Psychotherapeuten waren nicht ausreichend.
- ☐ Die Informationen der behandelnden Ärzte/Psychotherapeuten waren schwer zu verstehen.
- ☐ Ich war mit den Informationen der behandelnden Ärzte/Psychotherapeuten nicht einverstanden.
- ☐ Mir wurde vom Arzt/Psychotherapeuten empfohlen, mich über bipolare Störungen/Manien zu informieren.
- ☐ Ich bin auf der Suche nach einem Arzt/Psychotherapeuten.
- ☐ Ich wollte mich ganz allgemein über bipolare Störungen/Manien informieren.

- ☐ Sonstiges und zwar: (bitte angeben) [ ]

**Aus welchen Gründen haben Sie im Internet nach Informationen über somatoforme Störungen gesucht?**
Mehrfachangaben möglich

- ☐ Die Informationen der behandelnden Ärzte/Psychotherapeuten waren nicht ausreichend.
- ☐ Die Informationen der behandelnden Ärzte/Psychotherapeuten waren schwer zu verstehen.
- ☐ Ich war mit den Informationen der behandelnden Ärzte/Psychotherapeuten nicht einverstanden.
- ☐ Mir wurde vom Arzt/Psychotherapeuten empfohlen, mich über somatoforme Störungen zu informieren.
- ☐ Ich bin auf der Suche nach einem Arzt/Psychotherapeuten.
- ☐ Ich wollte mich ganz allgemein über somatoforme Störungen informieren.
- ☐ Sonstiges und zwar: (bitte angeben) [ ]

**Aus welchen Gründen haben Sie im Internet nach Informationen über Schizophrenie/Psychose gesucht?**
Mehrfachangaben möglich

- ☐ Die Informationen der behandelnden Ärzte/Psychotherapeuten waren nicht ausreichend.
- ☐ Die Informationen der behandelnden Ärzte/Psychotherapeuten waren schwer zu verstehen.
- ☐ Ich war mit den Informationen der behandelnden Ärzte/Psychotherapeuten nicht einverstanden.
- ☐ Mir wurde vom Arzt/Psychotherapeuten empfohlen, mich über Schizophrenie/Psychose zu informieren.
- ☐ Ich bin auf der Suche nach einem Arzt/Psychotherapeuten.
- ☐ Ich wollte mich ganz allgemein über Schizophrenie/Psychose informieren.
- ☐ Sonstiges und zwar: (bitte angeben) [ ]

**Aus welchen Gründen haben Sie im Internet nach Informationen über Essstörungen gesucht?**
Mehrfachangaben möglich

- ☐ Die Informationen der behandelnden Ärzte/Psychotherapeuten waren nicht ausreichend.
- ☐ Die Informationen der behandelnden Ärzte/Psychotherapeuten waren schwer zu verstehen.
- ☐ Ich war mit den Informationen der behandelnden Ärzte/Psychotherapeuten nicht einverstanden.
- ☐ Mir wurde vom Arzt/Psychotherapeuten empfohlen, mich über Essstörungen zu informieren.
- ☐ Ich bin auf der Suche nach einem Arzt/Psychotherapeuten.
- ☐ Ich wollte mich ganz allgemein über Essstörungen informieren.
- ☐ Sonstiges und zwar: (bitte angeben) [ ]

**Aus welchen Gründen haben Sie im Internet nach Informationen über Angststörungen gesucht?**
Mehrfachangaben möglich

- ☐ Die Informationen der behandelnden Ärzte/Psychotherapeuten waren nicht ausreichend.
- ☐ Die Informationen der behandelnden Ärzte/Psychotherapeuten waren schwer zu verstehen.
- ☐ Ich war mit den Informationen der behandelnden Ärzte/Psychotherapeuten nicht einverstanden.
- ☐ Mir wurde vom Arzt/Psychotherapeuten empfohlen, mich über Angststörungen zu informieren.
- ☐ Ich bin auf der Suche nach einem Arzt/Psychotherapeuten.
- ☐ Ich wollte mich ganz allgemein über Angststörungen informieren.
- ☐ Sonstiges und zwar: (bitte angeben) [ ]

**Aus welchen Gründen haben Sie im Internet nach Informationen über schädlichen Alkoholgebrauch oder Alkoholabhängigkeit gesucht?**
Mehrfachangaben möglich

- ☐ Die Informationen der behandelnden Ärzte/Psychotherapeuten waren nicht ausreichend.
- ☐ Die Informationen der behandelnden Ärzte/Psychotherapeuten waren schwer zu verstehen.
- ☐ Ich war mit den Informationen der behandelnden Ärzte/Psychotherapeuten nicht einverstanden.
- ☐ Mir wurde vom Arzt/Psychotherapeuten empfohlen, mich über schädlichen Alkoholgebrauch/Alkoholabhängigkeit zu informieren.

- ☐ Ich bin auf der Suche nach einem Arzt/Psychotherapeuten.
- ☐ Ich wollte mich ganz allgemein über schädlichen Alkoholgebrauch/Alkholabhängigkeit informieren.
- ☐ Sonstiges und zwar: (bitte angeben) ______

**Aus welchen Gründen haben Sie im Internet nach Informationen über psychische Erkrankungen gesucht?**
Mehrfachangaben möglich

- ☐ Die Informationen der behandelnden Ärzte/Psychotherapeuten waren nicht ausreichend.
- ☐ Die Informationen der behandelnden Ärzte/Psychotherapeuten waren schwer zu verstehen.
- ☐ Ich war mit den Informationen der behandelnden Ärzte/Psychotherapeuten nicht einverstanden.
- ☐ Mir wurde vom Arzt/Psychotherapeuten empfohlen, mich über psychische Erkrankungen zu informieren.
- ☐ Ich bin auf der Suche nach einem Arzt/Psychotherapeuten.
- ☐ Ich wollte mich ganz allgemein über psychische Erkrankungen informieren.
- ☐ Sonstiges und zwar: (bitte angeben) ______

**Nach welcher Art von Informationen über Depressionen haben Sie gesucht?**
Mehrfachangaben möglich

- ☐ Allgemeine Informationen über Depressionen (Beschwerden, Ursachen, Verlauf der Erkrankung)
- ☐ Informationen über Behandlungsmöglichkeiten (z.B. Psychotherapie, Medikamente)
- ☐ Informationen über Heilungschancen, Risiken und Nebenwirkungen einzelner Behandlungsmöglichkeiten
- ☐ Informationen über Psychotherapeuten, Ärzte, Krankenhäuser (z.B. Wo kann ich mich behandeln lassen?)
- ☐ Informationen über Selbsthilfegruppen/Austausch mit Betroffenen/Erfahrungsberichte von Betroffenen
- ☐ Informationen für Angehörige
- ☐ Tipps zum Umgang mit der Erkrankung (z.B. Alltagsgestaltung, Selbsthilfe)
- ☐ Sonstiges und zwar: (bitte angeben) ______

**Nach welcher Art von Informationen über bipolare Störungen/Manien haben Sie gesucht?**
Mehrfachangaben möglich

- ☐ Allgemeine Informationen über bipolare Störungen/Manien (Beschwerden, Ursachen, Verlauf der Erkrankung)
- ☐ Informationen über Heilungschancen, Risiken und Nebenwirkungen einzelner Behandlungsmöglichkeiten
- ☐ Informationen über Psychotherapeuten, Ärzte, Krankenhäuser (z.B. Wo kann ich mich behandeln lassen?)
- ☐ Informationen über Selbsthilfegruppen/Austausch mit Betroffenen/Erfahrungsberichte von Betroffenen
- ☐ Informationen für Angehörige
- ☐ Tipps zum Umgang mit der Erkrankung (z.B. Alltagsgestaltung, Selbsthilfe)
- ☐ Sonstiges und zwar: (bitte angeben) ______

**Nach welcher Art von Informationen über somatoforme Störungen haben Sie gesucht?**
Mehrfachangaben möglich

- ☐ Allgemeine Informationen über somatoforme Störungen (Beschwerden, Ursachen, Verlauf der Erkrankung)
- ☐ Informationen über Behandlungsmöglichkeiten (z.B. Psychotherapie, Medikamente)
- ☐ Informationen über Heilungschancen, Risiken und Nebenwirkungen einzelner Behandlungsmöglichkeiten

- ☐ Informationen über Psychotherapeuten, Ärzte, Krankenhäuser (z.B. Wo kann ich mich behandeln lassen?)
- ☐ Informationen über Selbsthilfegruppen/Austausch mit Betroffenen/Erfahrungsberichte von Betroffenen
- ☐ Informationen für Angehörige
- ☐ Tipps zum Umgang mit der Erkrankung (z.B. Alltagsgestaltung, Selbsthilfe)
- ☐ Sonstiges und zwar: (bitte angeben) ____

**Nach welcher Art von Informationen über Schizophrenie/Psychose haben Sie gesucht?**
Mehrfachangaben möglich

- ☐ Allgemeine Informationen über Schizophrenie/Psychose (Beschwerden, Ursachen, Verlauf der Erkrankung)
- ☐ Informationen über Behandlungsmöglichkeiten (z.B. Psychotherapie, Medikamente)
- ☐ Informationen über Heilungschancen, Risiken und Nebenwirkungen einzelner Behandlungsmöglichkeiten
- ☐ Informationen über Psychotherapeuten, Ärzte, Krankenhäuser (z.B. Wo kann ich mich behandeln lassen?)
- ☐ Informationen über Selbsthilfegruppen/Austausch mit Betroffenen/Erfahrungsberichte von Betroffenen
- ☐ Informationen für Angehörige
- ☐ Tipps zum Umgang mit der Erkrankung (z.B. Alltagsgestaltung, Selbsthilfe)
- ☐ Sonstiges und zwar: (bitte angeben) ____

**Nach welcher Art von Informationen über Essstörungen haben Sie gesucht?**
Mehrfachangaben möglich

- ☐ Allgemeine Informationen über Essstörungen (Beschwerden, Ursachen, Verlauf der Erkrankung)
- ☐ Informationen über Behandlungsmöglichkeiten (z.B. Psychotherapie, Medikamente)
- ☐ Informationen über Heilungschancen, Risiken und Nebenwirkungen einzelner Behandlungsmöglichkeiten
- ☐ Informationen über Psychotherapeuten, Ärzte, Krankenhäuser (z.B. Wo kann ich mich behandeln lassen?)
- ☐ Informationen über Selbsthilfegruppen/Austausch mit Betroffenen/Erfahrungsberichte von Betroffenen
- ☐ Informationen für Angehörige
- ☐ Tipps zum Umgang mit der Erkrankung (z.B. Alltagsgestaltung, Selbsthilfe)
- ☐ Sonstiges und zwar: (bitte angeben) ____

**Nach welcher Art von Informationen über schädlichen Alkoholgebrauch/Alkoholabhängigkeit haben Sie gesucht?**
Mehrfachangaben möglich

- ☐ Allgemeine Informationen über schädlichen Alkoholgebrauch/Alkoholabhängigkeit (Beschwerden, Ursachen, Verlauf der Erkrankung)
- ☐ Informationen über Behandlungsmöglichkeiten (z.B. Psychotherapie, Medikamente)
- ☐ Informationen über Heilungschancen, Risiken und Nebenwirkungen einzelner Behandlungsmöglichkeiten
- ☐ Informationen über Psychotherapeuten, Ärzte, Krankenhäuser (z.B. Wo kann ich mich behandeln lassen?)
- ☐ Informationen über Selbsthilfegruppen/Austausch mit Betroffenen/Erfahrungsberichte von Betroffenen
- ☐ Informationen für Angehörige
- ☐ Tipps zum Umgang mit der Erkrankung (z.B. Alltagsgestaltung, Selbsthilfe)
- ☐ Sonstiges und zwar: (bitte angeben) ____

**Nach welcher Art von Informationen über psychische Erkrankungen haben Sie gesucht?**
Mehrfachangaben möglich

- ☐ Allgemeine Informationen über psychische Erkrankungen (Beschwerden, Ursachen, Verlauf der Erkrankung)
- ☐ Informationen über Behandlungsmöglichkeiten (z.B. Psychotherapie, Medikamente)
- ☐ Informationen über Heilungschancen, Risiken und Nebenwirkungen einzelner Behandlungsmöglichkeiten
- ☐ Informationen über Psychotherapeuten, Ärzte, Krankenhäuser (z.B. Wo kann ich mich behandeln lassen?)
- ☐ Informationen über Selbsthilfegruppen/Austausch mit Betroffenen/Erfahrungsberichte von Betroffenen
- ☐ Informationen für Angehörige
- ☐ Tipps zum Umgang mit der Erkrankung (z.B. Alltagsgestaltung, Selbsthilfe)
- ☐ Sonstiges und zwar: (bitte angeben) ____

**9 Entscheidungen I**

**Im Verlauf einer psychischen Erkrankung müssen oft verschiedene Entscheidungen getroffen werden, z.B. ob Ihr Angehöriger oder Freund/Bekannter Medikamente nimmt oder eine Psychotherapie macht.**
Wer soll bei den Beschwerden Ihres Angehörigen oder Freundes/Bekannten die medizinischen bzw. therapeutischen Entscheidungen treffen?

| nur mein Angehöriger bzw. Freund/Bekannter | hauptsächlich mein Angehöriger bzw. Freund/Bekannter | mein Angehöriger bzw. Freund/Bekannter und der Arzt/ Psychotherapeut | hauptsächlich der Arzt/ Psychotherapeut | nur der Arzt/ Psychotherapeut |
|---|---|---|---|---|
| ○ | ○ | ○ | ○ | ○ |

**10 Entscheidungen II**

**Musste Ihr Angehöriger bzw. Freund/Bekannter oder sein Arzt/Psychotherapeut schon einmal eine der folgenden Entscheidungen fällen?**
Bitte geben Sie dies für jede Frage an und schätzen Sie auch ein, wie schwierig diese Entscheidung war.

| | JA, die Entscheidung war SEHR SCHWIERIG. | JA, die Entscheidung war EHER SCHWIERIG. | JA, die Entscheidung war EHER EINFACH. | JA, die Entscheidung war SEHR EINFACH. | NEIN, er musste diese Entscheidung noch nicht treffen. |
|---|---|---|---|---|---|
| ob er sich ambulant oder in einer Klinik behandeln lässt | ○ | ○ | ○ | ○ | ○ |
| ob er einen Entzug macht oder nicht | ○ | ○ | ○ | ○ | ○ |
| ob er zunächst eine Zeit abwartet und beobachtet, ob die Beschwerden von selbst besser werden oder ob sie/er gleich mit der Behandlung beginnt | ○ | ○ | ○ | ○ | ○ |
| ob er Medikamente nimmt oder nicht | ○ | ○ | ○ | ○ | ○ |
| welches Medikament (Psychopharmaka) er nimmt | ○ | ○ | ○ | ○ | ○ |
| ob er ein anderes Medikament oder eine andere Dosis nimmt | ○ | ○ | ○ | ○ | ○ |
| ob er ein Medikament weiternimmt oder wieder absetzt | ○ | ○ | ○ | ○ | ○ |
| ob er zusätzlich zum Neuroleptikum ein Antidepressivum nimmt oder nicht | ○ | ○ | ○ | ○ | ○ |
| ob er die Medikation täglich als Tablette oder als Depotmedikation (Spritze, deren Wirkung mehrere Wochen anhält) nimmt | ○ | ○ | ○ | ○ | ○ |

| | | | | | |
|---|---|---|---|---|---|
| ob er ein phasenprophylaktisches Medikament (z.B. Lithium) nimmt oder nicht | ○ | ○ | ○ | ○ | ○ |
| ob er eine Psychotherapie macht oder nicht | ○ | ○ | ○ | ○ | ○ |
| ob er seine laufende Psychotherapie beenden soll oder nicht | ○ | ○ | ○ | ○ | ○ |
| ob er eine verhaltenstherapeutische, tiefenpsychologisch fundierte oder analytische Psychotherapie macht | ○ | ○ | ○ | ○ | ○ |

## 11 Entscheidungen IIa

**Musste Ihr Angehöriger bzw. Freund/Bekannter oder sein Arzt/Psychotherapeut schon einmal eine der folgenden Entscheidungen fällen?**

Bitte geben Sie dies für jede Frage an und schätzen Sie auch ein, wie schwierig diese Entscheidung war.

| | JA, die Entscheidung war SEHR SCHWIERIG. | JA, die Entscheidung war EHER SCHWIERIG. | JA, die Entscheidung war EHER EINFACH. | JA, die Entscheidung war SEHR EINFACH. | NEIN, er musste diese Entscheidung noch nicht treffen. |
|---|---|---|---|---|---|
| ob er zusätzlich zu seiner laufenden Psychotherapie Medikamente (Psychopharmaka) nimmt oder nicht | ○ | ○ | ○ | ○ | ○ |
| ob er zusätzlich zur medikamentösen Behandlung eine Psychotherapie macht oder nicht | ○ | ○ | ○ | ○ | ○ |
| ob er entweder eine Psychotherapie macht oder ein Medikament nimmt | ○ | ○ | ○ | ○ | ○ |
| ob er ein Selbsthilfebuch (Ratgeber) durcharbeitet oder nicht | ○ | ○ | ○ | ○ | ○ |
| ob er alternativmedizinische Angebote (z.B. pflanzliche Medikamente, Akupunktur, Entspannungsverfahren) in Anspruch nimmt oder nicht | ○ | ○ | ○ | ○ | ○ |
| ob er Sport macht oder nicht | ○ | ○ | ○ | ○ | ○ |

**12 Entscheidungen IIb**

Gibt es weitere Entscheidungen, die Sie im Verlauf der Erkrankung Ihres Angehörigen bzw. Freundes/Bekannten wichtig fanden?

**13 Entscheidungen III**

Wenn Sie an die oben genannten Entscheidungen denken: Wer hat beim letzten Arzt- bzw. Psychotherapeutengespräch Ihres Wissens nach die Entscheidung über die Behandlung getroffen?

| nur mein Angehöriger bzw. Freund/Bekannter | hauptsächlich mein Angehöriger bzw. Freund/Bekannter | mein Angehöriger bzw. Freund/Bekannter und der Arzt/ Psychotherapeut | hauptsächlich der der Arzt/ Psychotherapeut | nur der der Arzt/ Psychotherapeut |
|---|---|---|---|---|
| ○ | ○ | ○ | ○ | ○ |

**14 Anmerkungen**

Falls Sie noch Anmerkungen zu der Befragung haben, können Sie diese hier eintragen.

Herzlichen Dank für Ihre Mitarbeit! Sie haben nun alle Fragen beantwortet.
Können wir Ihre Daten in anonymer Form (siehe Datenschutzinformation) für unsere Auswertung verwenden?

- ○ Ja, ich habe alle Fragen sinnvoll beantwortet. Meine Angaben können für diese Auswertung verwendet werden.
- ○ Nein, ich wollte "nur mal gucken", nehme zum wiederholten Mal teil oder möchte nicht, dass meine Angaben ausgewertet werden.

**HINWEIS:**
Wenn Sie nun auf den Button "Weiter" klicken, ist die Befragung beendet.
Sie können dann keine weiteren Änderungen vornehmen.

## 15 Filter: Mindestalter

### 15.1 Mindestalter

Wir danken Ihnen herzlich für Ihre Bereitschaft zur Teilnahme.
Ihren Angaben entnehmen wir, dass Sie jünger als 18 Jahre sind.
An unserer Befragung können nur Personen teilnehmen, die älter als 18 Jahre sind.
Auf unserer Webseite www.psychenet.de können Sie sich jederzeit über das Projekt psychenet, häufige psychische Erkrankungen und Hilfsangebote informieren!

## 16 Filter: Ablehnung

### 16.1 Ablehnung

Sie haben die Teilnahme an unserer Befragung abgelehnt.
Auf unserer Webseite www.psychenet.de können Sie sich jederzeit über das Projekt psychenet, häufige psychische Erkrankungen und Hilfsangebote informieren!
Wenn Sie doch noch an der Befragung teilnehmen möchten, können Sie dies unter Befragung tun.

## 17 Endseite

Die Befragung ist jetzt beendet.
**Sehr herzlich möchten wir uns noch einmal für Ihre Teilnahme bedanken!**
